# Optimization of Electrospun Poly(caprolactone) Fiber Diameter for Vascular Scaffolds to Maximize Smooth Muscle Cell Infiltration and Phenotype Modulation

**DOI:** 10.3390/polym11040643

**Published:** 2019-04-09

**Authors:** Dae Geun Han, Chi Bum Ahn, Ji-Hyun Lee, Yongsung Hwang, Joo Hyun Kim, Kook Yang Park, Jin Woo Lee, Kuk Hui Son

**Affiliations:** 1 Department of Health Sciences and Technology, GAIHST, Gachon University, 155 Gaetbeol-ro, Yeonsu-ku, Incheon 21999, Korea; hdg1592@naver.com (D.G.H.); noirsky@naver.com (J.H.K.); 2 Department of Molecular Medicine, College of Medicine, Gachon University, 155 Gaetbeol-ro, Yeonsu-ku, Incheon 21999, Korea; cutemole@gmail.com (C.B.A.); totoro218@hanmail.net (J.-H.L.); 3 Soonchunhyang Institute of Medi-bio Science, Soonchunhyang University, Cheonan-si 31151, Korea; yshwang428@sch.ac.kr; 4 Department of Thoracic and Cardiovascular Surgery, Gil Medical Center, Gachon University College of Medicine, 21, Namdong-daero 774 Beon-gil, Namdong-gu, Incheon 21565, Korea; kkyypark@gilhospital.com

**Keywords:** electrospinning, vascular smooth muscle, vascular scaffold, infiltration, optimization

## Abstract

Due to the morphological resemblance between the electrospun nanofibers and extracellular matrix (ECM), electrospun fibers have been widely used to fabricate scaffolds for tissue regeneration. Relationships between scaffold morphologies and cells are cell type dependent. In this study, we sought to determine an optimum electrospun fiber diameter for human vascular smooth muscle cell (VSMC) regeneration in vascular scaffolds. Scaffolds were produced using poly(caprolactone) (PCL) electrospun fiber diameters of 0.5, 0.7, 1, 2, 2.5, 5, 7 or 10 μm, and VSMC survivals, proliferations, infiltrations, and phenotypes were recorded after culturing cells on these scaffolds for one, four, seven, or 10 days. VSMC phenotypes and macrophage infiltrations into scaffolds were evaluated by implanting scaffolds subcutaneously in a mouse for seven, 14, or 28 days. We found that human VSMC survival was not dependent on the electrospun fiber diameter. In summary, increasing fiber diameter reduced VSMC proliferation, increased VSMC infiltration and increased macrophage infiltration and activation. Our results indicate that electrospun PCL fiber diameters of 7 or 10 µm are optimum in terms of VSMC infiltration and macrophage infiltration and activation, albeit at the expense of VSMC proliferation.

## 1. Introduction

Populations with cardiovascular disease are growing in many countries and their health care cost has also increased rapidly [1,2]. To cure the disease, replacements of damaged blood vessels by auto-transplantation are required, but most of the patients cannot supply suitable blood vessels for the surgery. Therefore, demands for non-autologous vessels have been increased and in the case of large-diameter (>6 mm) blood vessels, various synthetic vascular grafts were developed and well utilized. However, synthetic vascular grafts of diameter < 6 mm are frequently occluded by thrombosis, aneurysms, or intimal hyperplasia [3,4]. Tissue engineering provides an alternative approach in which cells are seeded or encapsulated in scaffolds fabricated from biodegradable polymers [3,5,6]. Until now, organs including bladder and trachea as well as tissues including bone, cartilage, skin and muscle, were regenerated by utilizing various types of biodegradable polymers [7,8,9,10,11,12]. Especially, the success of tissue engineered vascular grafts is governed, among other factors, by the development of a scaffold that mimics extracellular matrix (ECM), which is a 3D network of 50–500 nm diameter structural protein and polysaccharide fibers [13].

Morphological similarities between electrospun nanofibers and ECM are a major driver for the use of electrospun mats as a scaffold, and the high surface area: Volume ratios and interconnected pores of these fibrous meshes ensure cell attachment and oxygen/nutrient transport [14,15,16,17]. However, electrospinning has its limitations, such as, poor cellular infiltration [17,18]. Electrospun scaffolds consist of closely packed nanofiber layers that only provide a superficial porous structure, due to their sheet-like nature. Decreasing electrospun fiber diameters increases the number of fiber–fiber contacts/unit length, which reduces the average pore size [19]. Furthermore, cell infiltration is extremely important for tissue engineering scaffolds, since tissue regeneration cannot be achieved if cells do not proliferate inside scaffolds [20].

Vascular structures consist of three layers, that is, intima, media and adventitia [21]. The media consists of vascular smooth muscle cells (VSMCs) and provide mechanical strength and the vasoactive responsiveness of blood vessels [22,23,24]. Therefore, vascular grafts that facilitate VSMC penetration deep into grafts are a prerequisite for producing an integral media layer that mimics the function of the vascular smooth muscle [25].

VSMCs have either contractile or synthetic phenotypes. VSMCs of the synthetic phenotype can rapidly proliferate and produce ECM, whereas those of the contractile phenotype maintain the function of vascular media. Furthermore, it is critical that the contractile VSMC phenotype be achieved at the proper development stage, otherwise uncontrolled proliferation of VSMCs in grafts will thicken vessel walls and narrow lumen [25,26]. In addition, it is known that the mean scaffold pore size significantly affects cell morphology and phenotypic expressions [27].

This study was performed to determine the optimal poly(caprolactone) (PCL) electrospun fiber diameter that maximizes VSMC survival, proliferation, and infiltration and that modulates VSMC phenotypes in a manner compatible with those required for the development of vascular grafts. 

## 2. Materials and Methods

### 2.1. 3D Printed Support Layer

**Figure 1:**
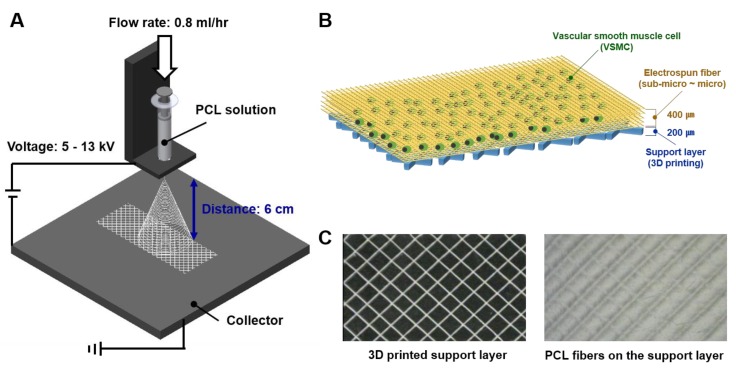
Fabrication process and results of the electrospinning based scaffold. (**A**) Schematic of electrospinning device and fiber fabrication process; (**B**) schematic of electrospun scaffold for vascular smooth muscle cells (VSMCs) culture; (**C**) shape of the 3D printed supporting layer and scaffold of electrospun fibers on the 3D printed supporting layer.

The scaffold for VSMC, which is only made with PCL fiber, was so thin and it was hard to handle for the VSMC culture. Thus, we made a support layer for PCL electrospun fiber deposition by a 3D printer (Geo technology, Incheon, Korea). The 3D printed support layer was the size of 150 mm (W) × 150 mm (L) × 0.2 mm (H) which consists of 2 × 2 mm sized squares and the material for printing was PCL (MW 45,000, Sigma-Aldrich, St. Louis, MO, USA) (Figure 1A,B).

### 2.2. PCL Electrospinning for PCL Fiber

Electrospun fibers were deposited on the 3D printed supporting layer using an electrospinning device (NanoNC, Seoul, Korea). Figure 1C showed fabrication conditions used by the diameter. The flow rate of the electrospinning solvent was fixed at 0.8 mL/h and the spinning distance was 60 mm. Fiber morphologies in scaffolds were observed using an optical microscope (Optical microscope, OPTIKA, Ponteranica (BG), Italy) and average fiber diameters were measured.

### 2.3. Cell Culture

Human VSMCs were purchased from the American Type Culture Collection (ATCC; No.CRL-1999, Manassas, VA, USA). Cells were cultured in the DMEM/F12 medium (Gibco, Waltham, MA, USA) containing 15% fetal bovine serum (FBS; Gibco, Waltham, MA, USA), 100 units/mL of penicillin/streptomycin (P/S; Gibco, Waltham, MA, USA) at 37 °C in a humidified 5% CO_2_ atmosphere. The medium was changed every 2–3 days. When cells reached confluence, they were removed from the culture dish using 0.25% trypsin-Ethlenediaminetetraacetic acid (EDTA; Gibco, Waltham, MA, USA), centrifuged, and resuspended in DMEM/F12. Scaffolds were soaked overnight in 70% EtOH, repeatedly rinsed with ultra-pure water, sterilized by exposing them to ultraviolet (UV) light for 30 min and then coated with 1% gelatin for 30 min before introducing the cells.

### 2.4. Live/Dead Assay

VSMCs were seeded at a density of 1.5 × 10^4^ cell/scaffold in a 96-well plate for one, four, seven or 10 days. The live/dead fluorescent solution of calcein-acetoxymethyl (AM) and ethidium homodimer-1 (EthD-1) was prepared according to the manufacturer’s instructions (Thermo Fisher, Waltham, MA, USA). Scaffolds were then submerged in the solution and incubated at 37 °C for 40 min before being washed in phosphate-buffered saline (PBS; Gibco, Waltham, MA, USA) and observed under a fluorescent microscope (Zeiss LSM 510, Oberkochen, Germany). Image J software was used to quantify calcein-AM staining levels in scaffolds to assess cell viability. Percentages of live cells were calculated by Equation (1).

Cell viability (%) = (number of live cells/number of total cells) × 100 (%)(1)

### 2.5. Cell Proliferation Assay

Cell proliferation rates were measured using a cell counting kit (CCK8; Dojindo, Kumamoto, Japan). Cells were seeded in 12-well plates at a density of 1.6 × 10^5^ cell/scaffold. After one, four, seven or 10 days, 100 µL of CCK-8 solution was added to each well and cells were incubated at 37 °C for 2 h. Absorbances were measured at 450 nm using an ELISA reader (VERSAmax, San Jose, CA, USA).

### 2.6. Immunocytochemistry

For the immunocytochemical analysis, VSMCs were seeded at a density of 1.5 × 10^4^ cell/scaffold in a 96-well plate for one, four, seven and 10 days. Cells were then washed twice with PBS, fixed for 15 min in PBS containing 4% paraformaldehyde (PFA; Bioworld, Gyeong gi-do, Korea) and gently washed three times with PBS. After fixation, cells were incubated for 10 min in a freezer in ice-cold 100% methanol (Sigma, St. Louis, MI, USA) and then rinsed with PBS for 5 min. Blocking was achieved by incubating cells for 60 min in 5% normal goat serum (Vector laboratories, Burlingame, CA, USA) in PBS/0.3% Triton X-100 (Sigma, St. Louis, MI, USA). VSMC seeded scaffolds were incubated overnight at 4 °C with alpha-smooth muscle actin (α-SMA) or non-muscle heavy chain antibodies (Abcam, Cambridge, UK) at a dilution of 1:100, washed twice in PBS, and incubation for 2 h with donkey mouse alexafluor 488-conjugated or donkey rabbit alexafluor 568-conjugated secondary antibodies (diluted at 1:500 in blocking solution; Abcam) in the dark. After washing cells with tris-buffered saline (TBS) twice, scaffolds were counterstained with 4’,6-diamidino-2-phenylindole (DAPI (to visualize nuclei; Vector Laboratories). Samples were then coverslipped and visualized and photographed under a fluorescent microscope.

### 2.7. Cell Infiltration

On days four, seven and 10 of culture, scaffolds were fixed in 4% PFA, stained with DAPI and cell infiltration was assessed by the fluorescent microscopy. The image J software was used to analyze the distribution of DAPI stained areas in each section. We defined the infiltration distance as the maximum distance travelled by VSMCs from the seeding surface. Three sections of each scaffold were used to quantify cell infiltration.

### 2.8. Experimental Animals

All protocols were approved by the Animal Subjects Committee of Gachon University. (Approval #: LCDI-2018-0002) Male mice (C57BL6, six weeks aged, weight: 30 g; Orientbio) were used. Mice were kept under controlled SPF conditions (22 to 24 °C, 55% to 60% RH) under a 12-h light/dark cycle. During the experimental period, mice were given free access to water and to a standard rodent diet. A scaffold containing 1.6 × 10^5^ VSMCs was then implanted into a subcutaneous pocket in the dorsum of each mouse. To suppress the immune rejection 5 mg/kg of cyclosporine (Sigma, St. Louis, MI, USA) was injected subcutaneously every day after scaffold implantation until scaffold harvest. Animals were sacrificed on days seven, 14 or 28.

### 2.9. Histology

Immediately after animal sacrifice, scaffolds were collected, fixed overnight in 4% PFA, dehydrated, embedded in paraffin and cut into 4 µm sections. Immunocytochemistry was performed using α-SMA or non-muscle heavy chain antibodies (diluted at 1:100). Alexa Fluor anti-mouse 488 or Alexa Fluor anti-rabbit 568 (diluted at 1:500) were used as secondary antibodies and nuclei were counterstained with DAPI. To determine activated macrophage expression levels, immunohistochemistry was performed using a peroxidase immunohistochymistry (IHC) detection kit (Thermo Fisher, Waltham, MA, USA) and Iba1 antibody (Abcam, Cambridge, UK) at a dilution of 1:100. Washed sections were treated with biotinylated horse anti-goat antibody (diluted at 1:200; Abcam) and then incubated in streptavidin conjugated with peroxidase. Staining was detected using 3,3-diaminobenzidine (DAB). All images were captured at a magnification of 15 ×.

### 2.10. Statistical Analysis

Statistical analysis was performed using the SPSS version 21 (IBM Corporation, Armonk, NY, USA). The significances of differences were determined by the one-way analysis of variance (ANOVA). Bonferroni’s multiple comparison test was used to investigate relations between variables and fiber diameters. *p* values of < 0.05 were deemed significant. Results are presented as means ± standard deviations.

## 3. Results

### 3.1. Fabrication of Scaffolds with Different Microstructures and Physical Properties

**Figure 2:**
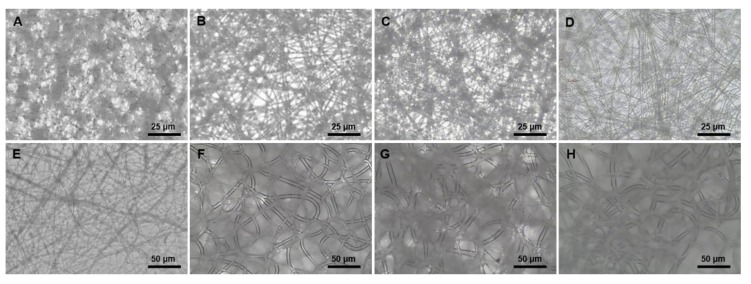
Morphologies of electrospun fibers. (**A**) 0.5 μm (diameter); (**B**) 0.7 μm (diameter); (**C**) 1 μm (diameter); (**D**) **2** μm (diameter); (**E**) 2.5 μm (diameter); (**F**) 5 μm (diameter); (**G**) 7 μm (diameter); (**H**) 10 μm (diameter).

To evaluate a cell response by various microstructures, we fabricated electrospun fiber scaffolds. After preparing the 3D printed PCL support of 150 mm (W) × 150 mm (L) × 0.2 mm (H) which consists of 2 × 2 mm sized squares, various size of PCL fibers were electrospun on the PCL support. The vertical thickness of electrospun layer was 400 ± 1 μm and their fibers had uniform circular shapes. In each scaffold, average diameters were 0.5, 0.7, 1, 2, 2.5, 5, 7, or 10 μm (Figure 2A ~ H). Mean pore sizes of the scaffolds ranged from 2.03 ± 0.32 μm to 16.97 ± 5.83 μm and pore sizes were increased with fiber diameter (Table 1).

### 3.2. In Vitro VSMC Survival on Scaffolds

**Figure 3:**
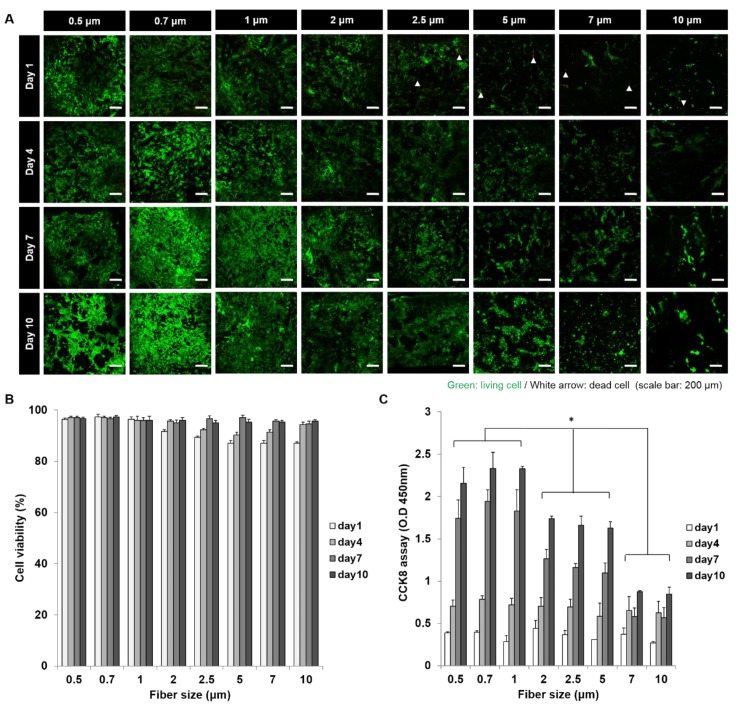
In vitro VSMC proliferation results in scaffolds. (**A**) Live/dead assay (Living cells (green), dead cells (red)); (**B**) cell survival rates in scaffolds with various fiber diameters; (**C**) cell proliferation results in scaffolds with various fiber diameters. (*: *p* < 0.05).

To estimate biocompatibility scaffolds, we observed the VSMC survival on each of the scaffolds with 8 types of fiber diameters using a live/dead assay (Figure 3A,B). During the VSMC culture time of 10 days, all scaffolds showed the cell survivals of more than 85% and they kept cell survival rate of 95% from day seven. Compared with scaffolds with various fiber sizes, after one and four days of culture, percentages of live cells on smaller fibers (95%) were greater than on larger fibers (85%). However, no difference was observed after seven or 10 days of culture. 

### 3.3. In Vitro VSMC Proliferation on Scaffolds

To evaluate the cell response of scaffolds with various pore and fiber sizes, we observed the VSMC proliferation using a CCK-8 assay (Figure 3C). During the first four days of culture, no significant relation was observed between proliferation and fiber diameter. However, after seven days, a proliferation rate of scaffolds with 0.5, 0.7 and 1 μm fibers (A, B, and C) was rapidly increased and scaffolds with 2, 2.5 and 5 μm (D, E, and F) fibers showed a moderate cell proliferation level. And at day 10, scaffolds with large fiber diameters of 7 and 10 μm (G and H) showed lowest proliferation performance, although a number of cells were increased.

### 3.4. In Vitro VSMC Infiltration

**Figure 4:**
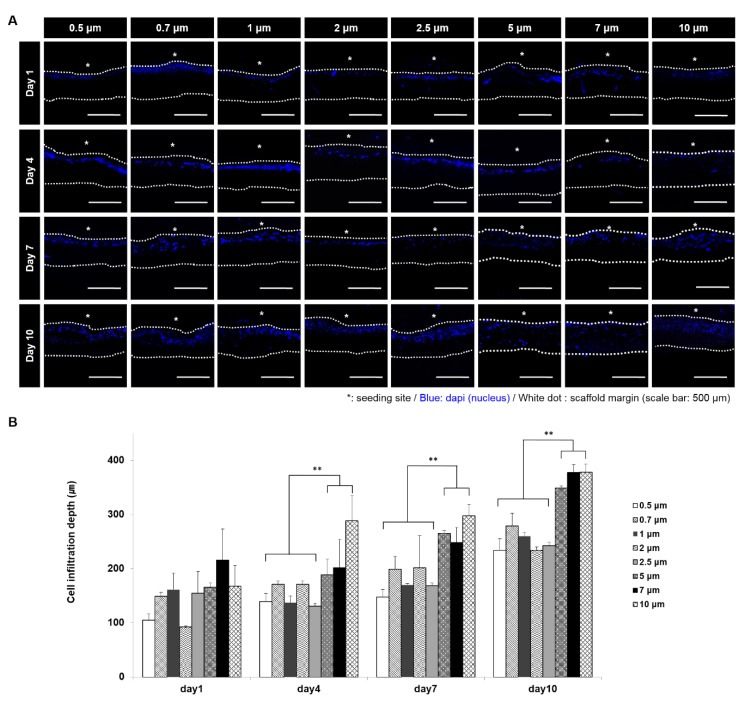
VSMC infiltration results in scaffolds. (**A**) DAPI staining results to determine infiltration depth. (White dotted line: scaffold margin. * (white): cell seeding site); (**B**) measurement results of VSMCs infiltration depth. (**: *p* < 0.01)

To evaluate the cell infiltration at scaffolds with various pore and fiber sizes, cross-sections of VSMC cultured scaffolds were analyzed by DAPI staining (Figure 4A,B). Infiltration distance increased as the fiber diameter increased, and was greatest for 5, 7, 10 μm fiber diameter scaffolds after four, seven, and 10 days of culture. Especially, at day 10, VSMCs were fully infiltrated on 5, 7, 10 μm fiber diameter scaffolds, while they showed an infiltration of a half of scaffold thickness on the other conditions.

### 3.5. Maintenance of VSMC Phenotype on Scaffolds

**Figure 5:**
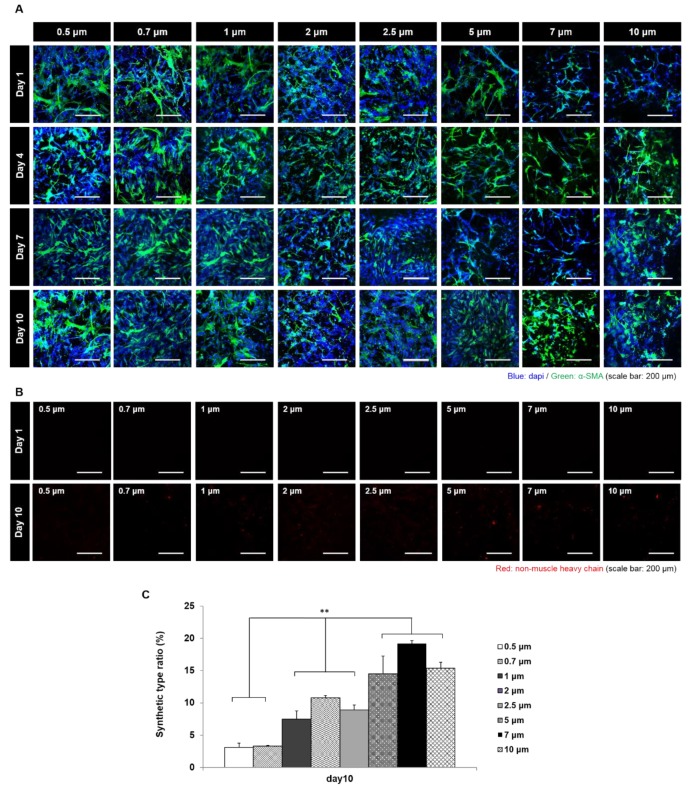
Analysis of the VSMC phenotype change in scaffolds (in vitro). (**A**) Staining results of VSMC phenotype change in scaffolds with various fiber diameters (red = Synthetic type VSMCs stained for non-muscle heavy chain, green = contractile type VSMCs stained for α-SMA); (**B**)comparison of the number of synthetic type VSMCs between culture day one and day 10; (**C**) calculation of phenotype change ratio of VSMCs in scaffolds. (**: *p* < 0.01)

We evaluated VSMC phenotypes on scaffold seeding surfaces (Figure 5A). The α-SMA stain was used to detect the contractile type and the non-muscle MHC was used to detect the synthetic type [28]. After culture for seven days, the contractile phenotype was maintained on all 8-scaffold types, but at 10 days the synthetic type started to appear (Figure 5B). When we defined ‘synthetic type ratio (%)’ as the ratio of synthetic type VSMC (non-muscle MHC positive cell) to total cells (DAPI positive) (Figure 5C), it was found that the change ratio increased with fiber diameter.

### 3.6. In Vivo Subcutaneous Implantation

**Figure 6:**
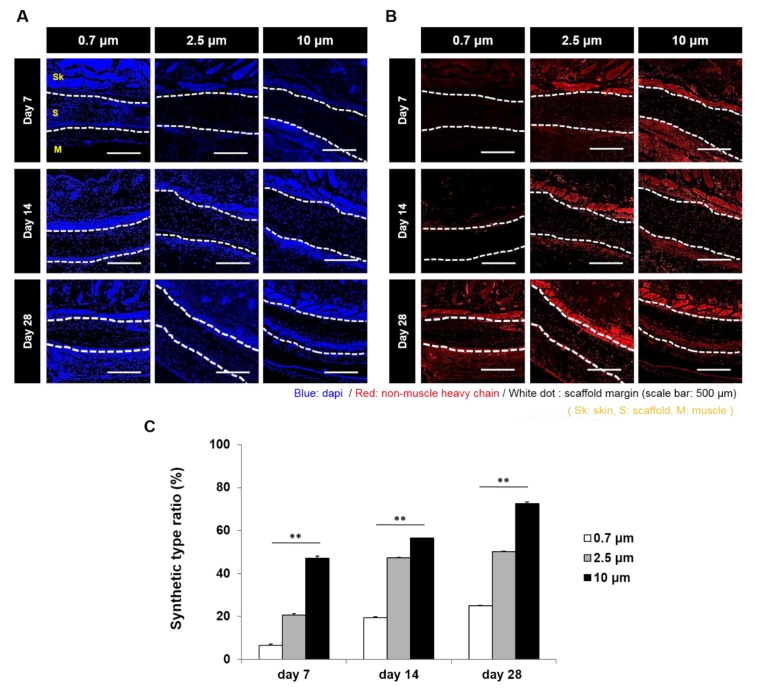
VSMC phenotype analysis in scaffolds implanted subcutaneously in mice. (**A**) Contractile type VSMCs stained with α-SMA; (**B**) synthetic type VSMCs stained with non-muscle heavy chain; (**C**) change of conversion ratio (from contractile type VSMCs to synthetic type VSMCs) at various fiber diameters (**: *p* < 0.01).

**Figure 7:**
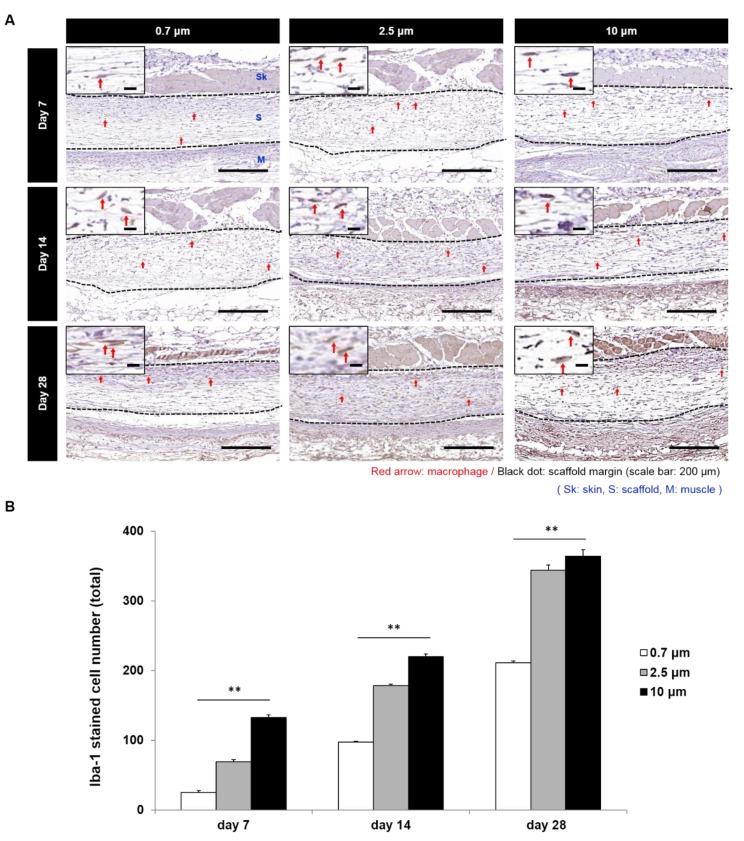
Macrophage activation in scaffolds with various fiber diameters. (**A**) Result of Iba 1 stain for activated macrophages. (Red arrow: Iba1 stained macrophage); (**B**) comparison of the number of activated macrophages in scaffolds with various fiber diameters. (**: *p* < 0.01).

To evaluate a phenotype change of SMC by the electrospun fiber diameter at in vivo, VSMC seeded 0.7, 2.5, and 10 μm scaffolds were implanted under the skin of mice (Figure 6A,B). Scaffolds with 0.7 μm fibers showed the lowest change ratio of the synthetic type VSMC and scaffolds with 10 μm fibers showed the highest value of the synthetic type VSMC at all time points. Namely, change ratios were found to increase with fiber diameter. In addition, when numbers of activated macrophages in scaffolds were assessed by Iba-1 staining, it was found that the activated macrophage numbers increased with fiber diameter at all time points (Figure 7A,B).

## 4. Discussion

Until now, various efforts have been made to improve cell adhesion, proliferation and differentiation by using electrospinning. However, there have been few studies on the effect of dividing the step from nano to micro-size sections for VSMC [29,30,31,32,33]. Therefore, in this study, we sought to identify an optimal diameter for PCL electrospun fiber with respect to VSMC proliferation, infiltration, and phenotype modulation in tubular scaffolds. VSMCs play important roles in vessels as they provide structural support and contractile function, and thus, the preparation of a mature smooth muscle layer on vascular scaffolds is required prior to implantation [34].

Intima is composed of endothelial cells (ECs), and ECs can proliferate rapidly on the luminal surfaces of electrospun scaffolds and cover luminal surfaces soon after seeding in vitro [20]. However, it remains a challenge to induce VSMCs to pass through the luminal surface and immigrate into the interior of a scaffold to proliferate and form a VSMC layer [35]. For this reason, we considered that we should start with a scaffold design optimized for SMC layer generation.

Cellular infiltration and other factors, such as, the diffusions of metabolites, nutrients, and waste, are often limited by the small pore sizes of electrospun scaffolds. Furthermore, pore size has been associated with cellular activity in many different cell types [36,37]. In the present study, cell survivals on scaffolds after seven and nine days of culture were not found to be fiber diameter dependent, but after one and four days of culture survivals were lower for larger fiber diameters, which were attributed to different infiltration differences due to the larger pores of larger fiber diameter scaffolds. Previous studies have shown that cell infiltration increases linearly with fiber diameter, and similarly, we observed the VSMC infiltration after one, four, seven, and 10 days of culture increased by fiber diameter [8]. In a previous study, the best cellular infiltration was achieved when pore size was close to that of target cells, which suggests that optimum pore size is cell-specific [18,19]. In terms of cell infiltration, we found that fiber diameters of 7 or 10 µm were optimum in terms of maximizing the human VSMC infiltration.

As opposed to infiltration, proliferation of SMCs has been previously reported to be inversely related to fiber diameter, and our findings concur [3]. VSMCs regulate their phenotype in response to environmental chemical, physical, and mechanical signals, and under normal physiological conditions VSMCs rarely proliferate. However, these cells can grow rapidly under some pathologic conditions, such as, atherosclerosis [38]. The vascular regeneration of contractile VSMCs on scaffolds may facilitate the production of functional tissue-engineered blood vessels, and their proliferation on scaffolds is essential for generating vascular tissues. However, the uncontrolled proliferation of VSMCs in implanted grafts causes the thickening of vessel walls and intimal hyperplasia and narrowing of vessel lumens [38,39]. Thus, VSMCs that infiltrate scaffolds should exhibit appropriate phenotypes at specific times. During the early stage, infiltrated VSMCs should exhibit the synthetic phenotype in order to complete the construction of a smooth muscle layer, whereas later they should express the contractile phenotype to produce a layer with normal psychological functions, which included providing mechanical strength and maintaining the integrity of vascular grafts [40].

Previous studies have reported that seeded VSMCs display a strongly contractile phenotype, and that after prolonged culture they exhibit a predominantly synthetic phenotype [41,42]. Similarly, in the present study, VSMCs seeded on scaffolds and cultured in vitro retained the contractile phenotype for 10 days when the synthetic phenotype was first observed. Furthermore, the number of synthetic VSMCs was found to increase with fiber diameter. Interestingly, our in vivo study showed a similar trend, as at seven, 14, and 28 days after scaffold implantation, the synthetic VSMC numbers increased with the fiber diameter. In a previous study, the contractile type of VSMCs were observed three months after the implantation of electrospun poly(glycerol sebacate) (PGS)-PCL vascular grafts in rat aortas [43].

The VSMC layer formation in vascular grafts takes considerable time and the synthetic type VSMCs would be expected to play an important role during the VSMC layer regeneration phase, and during the later remodeling phase, synthetic VSMCs should adopt the contractile phenotype. In our opinion, the in vivo study for 28 days shows a meaningful increase of synthetic phenotype for VSMC layer regeneration. Unfortunately, we did not extend our in vivo observations beyond 28 days, and thus, did not confirm the later synthetic to contractile phenotype shift and cannot comment on the effect of fiber diameter on the VSMC type modulation. Nonetheless, we did find that the larger fiber diameter scaffolds are probably advantageous in terms of the initial VSMC layer regeneration.

Macrophage activation is induced under pro-inflammatory and inflammatory conditions [44]. During the wound healing process, macrophage activation is essential. In the early wound, monocytes and resident macrophages become activated, undertake phagocytosis of microbes and perhaps early neutrophils, and produce pro-inflammatory mediators and chemo-attractants [45]. Macrophages also assist in the induction of apoptosis in neutrophils, thus turning the wound towards a non-inflammatory, reparative state. In the later phases of wound repair, macrophages ingest apoptotic neutrophils, producing growth factors to support tissue restoration. In the very late stages, as the wound resolves, macrophages may guide tissue remodeling by producing factors to promote capillary regression and collagen remodeling [45].

It is known that macrophages are able to infiltrate scaffolds with large pores more easily, and that proliferating macrophages secrete angiogenic factors that stimulate neovascularization in adventitia and maintain a high number of capillaries in the synthetic scaffolds, which is helpful for recruiting sufficient myofibroblasts to form a natural ECM in the scaffold walls [46,47,48,49]. In the present study, Iba1 stained activated macrophages were more prominent in the larger fiber diameter scaffolds, and it seemed that larger fiber scaffolds were better in terms of stimulating neovascularization by macrophages. In the present study, Iba1 which was used to label activated macrophages [50], were more prominent in the larger fiber diameter scaffolds, and it seemed that larger fiber scaffolds were better in terms of stimulating neovascularization by macrophages.

Scaffold characteristics have a strong influence on in situ tissue regeneration. When cells interact with a scaffold, they sense both the material (ionic and electrostatic interactions) and the microarchitecture (local geometry—film, fibers, spheres, sponge; porosity, pore size, and local compliance [51]. Designing scaffolds to direct the cell morphology, and, therefore, mechanics, is dependent on the typical cell dimensions in target tissues [52,53]. Our study shows that the human VSMC survival was not dependent on the electrospun fiber diameter. Cell proliferation decreased with scaffold fiber diameter, cells infiltration increased with fiber diameter, and numbers of VSMCs of the synthetic phenotype and of activated macrophages in scaffolds increased with fiber diameter. Our findings indicate that electrospun fiber diameters of 7 to 10 µm are better than smaller fiber diameters during the early medial layer regeneration phase in terms of the VSMC layer regeneration in scaffolds as VSMC infiltration, adoption of the synthetic VSMC, and increased macrophage numbers, albeit at the expense of VSMC proliferation. 

## Figures and Tables

**Table 1:** Electrospun fiber diameters and their fabricating conditions.

Condition	Fiber Diameter (μm)	Concentration of PCL (wt%)	Chloroform/Methanol	Voltage (kV)	Pore Size (μm)
**A** (0.5 µm)	0.53 ± 0.09	5	1:1	5	2.03 ± 0.32
**B** (0.7 µm)	0.69 ± 0.07	7.5	1:1	8	4.51 ± 1.49
**C** (1 µm)	1.01 ± 0.03	7.5	3:1	13	6.06 ± 1.58
**D** (2 µm)	1.98 ± 0.07	7.5	4:1	13	6.76 ± 1.97
**E** (2.5 µm)	2.51 ± 0.57	7.5	4:1	9	7.15 ± 1.26
**F** (5 µm)	5.06 ± 0.05	7.5	100:0	8	16.66 ± 5.20
**G** (7 µm)	7.05 ± 0.68	10	100:0	7.5	16.40 ± 4.96
**H** (10 µm)	10.16 ± 0.76	10	100:0	7	16.97 ± 5.83
